# Acute Kidney Injury in Children: Classification, Recognition and Treatment Principles

**DOI:** 10.3390/children11111308

**Published:** 2024-10-29

**Authors:** Matjaž Kopač

**Affiliations:** Department of Nephrology, Division of Pediatrics, University Medical Centre Ljubljana, Bohoričeva 20, 1000 Ljubljana, Slovenia; matjaz.kopac@siol.net; Tel.: +386-15229626

**Keywords:** acute kidney injury, children, diuretics, urine output, hypervolemia, electrolytes, acid–base balance, kidney perfusion

## Abstract

Acute kidney injury (AKI) in children is a critical medical condition characterized by a sudden decline in kidney function. This article provides a comprehensive overview of AKI in pediatric populations, exploring its pathophysiology, the role of various drugs and the long-term implications for kidney health. Key topics include oliguria, anuria, urine output, hypervolemia and the interactions among them, as well as role of diuretic nephrotoxicity and the glomerular filtration rate. Concepts of electrolytes, acid–base balance and renal perfusion assessment are presented. Basic principles of intensive care unit (ICU) management, renal replacement therapy and the association with multiorgan failure are described. Additionally, the article discusses the potential long-term outcomes of AKI, including the risk of chronic kidney disease, hypertension and proteinuria.

## 1. Introduction

Acute kidney injury (AKI) is a severe and potentially life-threatening condition characterized by a rapid decline in kidney function. While historically considered an adult-centric concern, AKI is increasingly recognized as a significant issue in pediatric populations. This article aims to explore the pathophysiology of AKI in children, focusing also on recognition and the role of various drugs in its management and the long-term implications for kidney health. AKI in children is defined by a sudden decrease in kidney function and is often assessed through parameters like serum creatinine and urine output. The Kidney Disease: Improving Global Outcomes (KDIGO) classification system categorizes AKI based on these criteria, offering a standardized approach to diagnosis and management [1]. It is important to know that serum creatinine is often a late and imprecise test of renal function because it reflects the glomerular filtration rate (GFR) in individuals in a steady state with stable kidney function. Therefore, it does not precisely reflect the GFR in a patient with a changing kidney function. In addition, serum creatinine levels depend upon various extrarenal factors, such as the age, sex, muscle mass, associated diseases and nutritional and hydration statuses of the child. Nevertheless, an elevated serum creatinine level is the most common laboratory parameter used for the diagnosis of AKI in children. However, the degree of oliguria affects fluid and electrolyte management and has a strong correlation with poor outcomes in children with AKI. Therefore, urine output measurement is very useful, particularly in the critical care setting. But, on the other hand, the presence of a normal volume of urine does not exclude AKI [2]. Oliguria, which is characterized by reduced urine output, is a hallmark of AKI. Anuria, the absence of urine production, represents a severe form. Both oliguria and anuria signify impaired renal function and inadequate filtration, necessitating prompt intervention [3].

Standardized and widely accepted definitions for pediatric AKI include the KDIGO (Kidney Disease Improving Global Outcomes) and pRIFLE (Pediatric Risk, Injury, Failure, Loss, End-Stage Renal Disease) classifications. The KDIGO AKI definition and staging have been accepted by the pediatric nephrology community in order to guide clinical care as well as provide standardized measures in AKI pediatric studies. Table 1 presents these definitions [2].

The purpose of this review is to present the important and complex issue of acute kidney injury to pediatricians from various subspecialties, such as general pediatricians, who usually make the first contact with these children, as well as to those working in intensive care units. This is especially important regarding the increased mortality that is associated with it, mostly in critically ill children and in those in need of renal replacement therapy (RRT). In addition, it appears, sometimes, that there is a lack of knowledge regarding acute kidney injury in children among healthcare professionals, especially in the context of multiorgan failure as well as in asymptomatic cases that often are unrecognized.

## 2. Classifications

Several AKI classifications have been developed. The most widely used is the one that separates the causes of AKI into the categories based on the anatomical location of the initial injury, which is helpful for understanding the predisposing pathophysiology and treatment approach. Table 2 presents the AKI classifications [2].

## 3. Clinical Diagnostic Evaluation

Diagnostic evaluation consists of a thorough history, physical examination, laboratory evaluation, imaging of the urinary tract and, rarely, a kidney biopsy. The aim of the history is to uncover a risk factor or cause for AKI, such as vomiting, diarrhea or decreased oral fluid intake, suggesting prerenal AKI. Bloody diarrhea before the onset of AKI is suggestive of hemolytic uremic syndrome, for example. Pharyngitis or, rarely, impetigo a few weeks prior to macrohematuria or edema is typical for poststreptococcal glomerulonephritis. Nephrotoxic drugs or hypotensive episodes are usually associated with intrinsic AKI, especially in hospitalized patients. But in patients with autoimmune diseases or vasculitides, such as IgA vasculitis (Henoch–Schönlein purpura) or systemic lupus erythematous, systemic signs may be present, such as fever, joint involvement or rash [2].

The physical examination includes a blood pressure measurement, assessment for edema and recent weight increase and signs of systemic disease. There are some physical findings, specific for underlying etiology, such as signs of volume depletion in prerenal AKI (dry mucosa, tachycardia and decreased skin turgor), edema in children with nephrotic syndrome or glomerulonephritis (the latter often accompanied by elevated blood pressure) and an enlarged bladder, suggestive of lower urinary tract obstruction. Daily assessment of body weight is crucial, especially for critically ill children, in whom hypervolemia with more than 10% increase in body weight compared to admission weight is associated with increased morbidity and mortality. Moreover, in children with AKI who need RRT and with fluid overload above 20%, mortality is increased above 700% [2]. In AKI, hypervolemia often occurs due to impaired fluid excretion. For this reason, diuretics, such as furosemide, are commonly employed to address fluid overload. However, their use requires careful consideration, as diuretic nephrotoxicity can exacerbate renal damage [4].

Laboratory studies used to determine the underlying etiology of AKI are urinalysis, blood tests and fractional sodium excretion. Table 3 presents these studies in more detail [2].

Biomarkers, including serum creatinine and urea, play a crucial role in diagnosing and monitoring AKI. Periodic assessments of renal function aid in treatment adjustments and provide insights into the progression or resolution of the condition [5]. Serum creatinine is the most common laboratory test used to detect a decreased GFR as a marker of AKI. However, it accurately reflects the GFR only in patients with stable renal function. When it is not known if the GFR is decreased according to the initial creatinine level, subsequent regular measurements revealing a rise in its concentration confirms the diagnosis of AKI. The serum creatinine can be detected only with at least a 50% reduction in the GFR in some cases. Some studies suggest that cystatin C has the potential to be a laboratory parameter that more accurately predicts AKI in children. It is freely filtered by the kidney with complete reabsorption and catabolism in the proximal tubules with negligible urinary excretion. Therefore, it is less affected by extrarenal factors compared to creatinine except for thyroid dysfunction, corticosteroids and inflammatory diseases. However, unlike creatinine, its measurements are expensive and are not widely available [2].

The ratio between serum blood urea nitrogen (BUN) and creatinine concentrations is significantly increased in patients with prerenal AKI (being over 20) and normal in patients with ATN (between 10 and 15). The reason for the difference is increased urea reabsorption by the proximal tubules, otherwise impermeable to creatinine. But increased BUN is present also in patients with increased urea production (administration of steroids; total parenteral nutrition), catabolic states and gastrointestinal bleeding, and therefore, this ratio has limited utility. In some patients, fluid administration of 10–20 mL/kg of normal saline might be both diagnostic and therapeutic. After such a fluid challenge, a decrease of BUN and serum creatinine suggests prerenal AKI, while a lack of their improvement, especially with simultaneous signs of fluid overload, is in favor of intrinsic AKI. Therefore, fluid administration is contraindicated in patients with established hypervolemia [2]. In addition, AKI significantly impairs the GFR, leading to electrolyte imbalances and disruptions in acid–base homeostasis. These alterations can contribute to complications and necessitate meticulous management to restore equilibrium [6].

Translational research using animal models of AKI has discovered various novel urinary proteins of kidney injury that are either secreted in urine due to renal tubular cell injury or are involved in AKI pathophysiology or cellular repair, enabling the early identification of tissue injury prior to function deterioration (detected by serum creatinine rise or urine output decrease). These AKI biomarkers can be classified into tubular damage biomarkers (mainly filtered urinary proteins normally reabsorbed by intact proximal tubular cells, such as beta-2 microglobulin or *N*-acetyl-beta-d-glucosaminidase), biomarkers whose genes are induced or downregulated by AKI (neutrophil gelatinase associated lipocalin (NGAL), interleukin-18 (IL-18) and kidney injury molecule-1 (KIM-1), for example) or biomarkers of cell cycle arrest (tissue inhibitor of metalloproteinases-2 (TIMP-2) and insulin-like growth factor binding protein-7 (IGFBP-7), for example) [7]. AKI biomarker research in children has focused mostly on urine NGAL, IL-18, KIM-1 and some other proteins. According to studies in children undergoing cardiac surgery and suffering from AKI for various reasons (treatment with chemotherapies or other nephrotoxins), these urine biomarker concentrations increase up to 48 h prior to serum creatinine and predict worse outcomes and morbidity. NGAL, the AKI biomarker with the strongest evidence for early detection of AKI in children, is already available for measurement using standard laboratory methods. However, current AKI biomarker use is mainly limited to research settings, but this may change in the future when they will probably be included in AKI definitions beside the established markers of kidney function, such as serum creatinine and urine output [7].

The excretion of endogenous and exogenous compounds and their metabolites is a key component of kidney function. Some of these substances are toxic to the kidney and can thus cause kidney injury and are, therefore, called nephrotoxins. They can occur either naturally or as pharmaceutical agents used to treat many diseases. It is important to understand the nephrotoxic potential of any given agent. This knowledge allows healthcare professionals to effectively manage both exposures and nephrotoxic injury. Although specific interventions for nephrotoxic AKI are not available yet, multidisciplinary strategies designed to increase awareness, reduce exposure and recognize injury have proven effective. There are several types of nephrotoxins, which are presented in Table 4. In addition, several different mechanisms of nephrotoxicity are presented in Table 5 [7].

Regarding imaging, a renal ultrasound is very useful in children with AKI of unclear etiology. It can detect the presence of renal agenesis, the renal size and can assess the parenchyma. In addition, it is very helpful in diagnosing a urinary tract obstruction or the occlusion of the major renal vessels and in differentiating AKI (where the size of the kidneys, with increased echogenicity, is normal or increased) from chronic kidney disease (usually small and shrunken) [2]. In addition, computer tomography (CT) or magnetic resonance (MR) imaging is sometimes indicated, especially in cases with suspected mass or kidney stones; the use of other imaging modalities depends on etiology and includes a voiding cystourethrogram (VCUG) and renal scintigraphy, either with a MAG3 scan or DMSA scan [7]. A systematic review of studies using contrast-enhanced ultrasonography (CEUS) to assess renal cortical microcirculation in AKI revealed that patients with AKI had reduced microcirculatory perfusion, a prolonged perfusion time and a reduced rising slope in the renal cortex, which occurred before serum creatinine changes. This indicates that CEUS could be helpful in the diagnosis of AKI [8].

## 4. Basic Principles of Pediatric AKI Treatment

Supportive measures include careful fluid management, the maintenance of electrolyte balance, and addressing the underlying cause of AKI. These measures are crucial for optimizing conditions for kidney recovery [9]. Adequate fluid administration in children with hypovolemia, the avoidance of hypotension in critically ill children by providing inotropic support if indicated and the adjustment of the dosing of nephrotoxic medications based on close monitoring of renal function and drug levels whenever possible are the most important measures for the prevention of AKI. Routine use of certain drugs (such as loop diuretics, mannitol, low-dose dopamine, fenoldopam and others) is not indicated for prevention of AKI in high-risk children. Table 6 presents the principles of the supportive measures in more detail [10].

In severe cases of pediatric AKI, especially when oliguria or anuria persists, RRT may be initiated. This can include modalities like hemodialysis or continuous renal replacement therapy (CRRT) to provide temporary renal support [11]. AKI is an independent predictor of morbidity and mortality, especially in critically ill children. RRT may prevent and correct the adverse and potentially life-threatening complications and improve survival. Urgent indications for RRT in children with AKI are clinically significant hypervolemia that is unresponsive to diuretic therapy (especially if associated with increased ventilatory requirements, usually in children with above a 15% degree of fluid overload), hyperkalemia and persistent metabolic acidosis (both unresponsive to medical treatment), complications of uremia (pericarditis, encephalopathy and bleeding) and exposure to toxins that are dialyzable and are inadequately excreted by the kidney. However, RRT should be initiated early according to the BUN level and before the development of clinical signs and symptoms of AKI rather than delaying dialysis until the child is symptomatic. It is indicated as well in critically ill children with oliguria despite diuretic therapy who require high volumes of intravenous fluids for drugs, nutrition or transfusions [12].

Several RRT modalities, including peritoneal dialysis (PD), intermittent hemodialysis and continuous RRT, are available to treat children with AKI. PD is the most common modality utilized in children, especially in infants and small children. There is not enough available data to favor one modality over another. Therefore, the selection of modality of RRT is based on patient characteristics (size, comorbidity and ability to obtain access), local expertise and experience and available resources. In addition, initiating RRT in a critically ill child requires collaboration among nephrologists and other different subspecialists caring for the child. Early discussion and planning will enhance the therapeutic process and help to improve the outcome. Retrospective data demonstrate the overall survival rates range between 50 and 75% in children with AKI who need RRT. Risk factors for mortality include the underlying disease, hypotension, marked hypervolemia at the initiation of RRT, use of inotropic therapy during RRT and age below one year [12].

## 5. Long-Term Implications of Pediatric AKI

AKI in childhood poses a heightened risk of developing chronic kidney disease (CKD) in later life. The severity and duration of AKI are correlated with the likelihood of CKD development, highlighting the importance of ongoing monitoring [13]. Children who have experienced AKI may be at an increased risk of developing hypertension as well. The disruption in renal function and hemodynamics during AKI may contribute to long-term blood pressure abnormalities [14]. Proteinuria, an abnormal presence of proteins in the urine, is another potential long-term consequence of AKI. This further underscores the need for ongoing monitoring and early intervention to mitigate the risk of progressive kidney damage [15]. In addition, AKI in children is associated with increased mortality, especially in critically ill children and in those in need of RRT. Children with moderate to severe AKI should be followed regularly (at least once a year) to detect signs of CKD, such as hypertension and proteinuria [10].

The KDIGO guidelines recommend that patients who develop AKI should be evaluated at three months after discharge to evaluate for recovery from AKI and the development of CKD [1]. No studies have specifically determined the adequate time or frequency for follow-up after an episode of AKI in children, especially regarding varying resource issues in different healthcare settings [7]. One approach suggests that children with Stage 2 or 3 AKI are identified using electronic health records, triggering referral and follow-up visits for CKD and hypertension screening and treatment at three, six and twelve months. However, this approach requires institutional support and multi-team coordination [16]. Another proposed approach is to risk stratify and select AKI patients who would benefit the most from follow-up according to AKI severity, non-recovery from AKI before discharge, the location of treatment (intensive care unit vs. hospital department), urinary biomarker profile and the presence of AKI recurrence [17]. It is important to ensure that patients at risk of CKD, baseline kidney disease or significant kidney dysfunction or hypertension before discharge are followed by a nephrologist after discharge. Previously healthy children with no risk factors mentioned previously and complete AKI recovery before discharge should undergo at least urine and blood pressure examinations at the first follow-up visit. If all parameters are normal and the patient is considered as low risk, a follow-up kidney health assessment approximately one year later, with reassessments every one or two years, is a reasonable approach [7].

## 6. Conclusions

Pediatric AKI is a critical condition that demands prompt recognition and intervention. The pathophysiology involves disruptions in kidney function, fluid balance and electrolyte homeostasis. Diuretics, while essential in managing fluid overload, should be used judiciously to avoid nephrotoxicity. In the intensive care unit, renal replacement therapy may be necessary for severe cases. The long-term implications of pediatric AKI, including the risk of chronic kidney disease, hypertension and proteinuria, underscore the importance of ongoing monitoring and early intervention to optimize outcomes. Further research and clinical advancements are essential to enhance our understanding and management of pediatric AKI.

In addition, AKI has been increasingly recognized globally as a common and important medical condition, with potentially long-term negative impacts on kidney function and other health-related outcomes. Several initiatives have started to carry out research and guide the care of children with AKI, with a focus on awareness by the patients, healthcare professionals and healthcare systems in order to improve AKI diagnosis, healthcare delivery and prognosis [7].

## Figures and Tables

**Table 1:** Criteria for KDIGO (Kidney Disease Improving Global Outcomes) definition of acute kidney injury (AKI) in children.

AKI Stage	Serum Creatinine	Urine Output
1	Increase in serum creatinine by ≥0.3 mg/dL from baseline (≥26.5 µmol/L) within 48 h or increase in serum creatinine to ≥ 1.5 times baseline within prior 7 days	Urine volume ≤ 0.5 mL/kg/h for six hours
2	Increase in serum creatinine to 2–2.9 times from baseline	Urine volume < 0.5 mL/kg/h for 12 h
3	Increase in serum creatinine to > 3 times from baseline or creatinine concentration > 353.6 µmol/L (4 mg/dL) or initiation of RRT or estimated GFR < 35 mL/min/1.73 m^2^	Urine volume < 0.3 mL/kg/h for 24 h or anuria for at least 24 h

Abbreviations: AKI—acute kidney injury; GFR—glomerular filtration rate; RRT—renal replacement therapy.

**Table 2:** Acute kidney injury classifications.

Causes of AKI		Main Characteristics
Anatomical location of the initial injury	Prerenal disease	The most common form of AKI in children
		Volume-responsive or functional AKI
		Decreased renal perfusion due to hypovolemia (bleeding, GI, urinary or cutaneous losses) or decreased effective circulation (heart failure, septic shock and cirrhosis)
		Reduced GFR and normal renal tubular function with increased reabsorption of Na^+^ and water, causing oliguria
		Urine flow and GFR return to normal after correction of renal perfusion
	Intrinsic kidney disease	Structural damage to the renal parenchyma
		Most commonly due to prolonged hypoperfusion, sepsis, nephrotoxins or severe glomerular diseases
	Postrenal disease	Usually due to congenital or acquired anatomic obstructions to the lower urinary tract
Clinical setting or circumstance	Community-acquired AKI	Associated with a single predominant insult, such as volume depletion
		Often reversible
	Hospital-acquired AKI	Usually in the critical care setting
		Multifactorial and part of multiorgan failure
		Profoundly complicates treatment and outcome
Urine output	Anuria	No urine output
	Oliguria	<1 mL/kg/h in infants <0.5 mL/kg/h in children and adults > 6 h
	Nonoliguria	>1 mL/kg/h in infants >0.5 mL/kg/h in children and adults > 6 h
	Polyuria	>3 mL/kg/h, often in patients with ATN and nephrotoxic AKI, with impaired urinary concentrating defect

Abbreviations: AKI—acute kidney injury; ATN—acute tubular necrosis; GFR—glomerular filtration rate; GI—gastrointestinal; Na^+^—sodium.

**Table 3:** Laboratory studies used in the evaluation process of acute kidney injury (AKI).

Laboratory Test	Main Characteristics of Specific Findings
*Urinalysis*	Muddy brown granular casts and epithelial cell casts suggest intrinsic AKI or ATN
	Red cell casts suggest glomerulonephritis, especially when associated with dysmorphic red cells and marked proteinuria; white cells and white cell casts may be present
	Pyuria with white cell, granular or waxy casts indicate tubulointerstitial disease or UTI
	A positive test for heme on a urine dipstick without red blood cells in the sediment suggest hemolysis or rhabdomyolysis
	Usually normal in cases with prerenal AKI
	Patients with ATN have σ < 1.010 and urine osmolality (more accurate measure of concentrating ability) < 350 mosmol/kg while those with prerenal AKI have σ > 1.020 and urine osmolality > 500 mosmol/kg
*Fractional excretion of Na^+^*	FE_Na_ < 1 (<2 in neonates): prerenal AKI, majority of the filtered Na^+^ is reabsorbed as a response to reduced perfusion
	FE_Na_ > 2 (>2.5 in neonates): indicates ATN
	FE_Na_ 1–2: inconclusive
	Limitations of FE_Na_: fluid administration prior to measurement, diuretic use and AKI due to contrast nephropathy or pigment nephropathy

Abbreviations: AKI—acute kidney injury; ATN—acute tubular necrosis; Na^+^—sodium; σ—urine specific gravity; UTI—urinary tract infection.

**Table 4:** Types of nephrotoxins.

Types of Nephrotoxins	Special Features
Pharmaceuticals	Many drugs (prescription or over the counter) have a side effect profile with potential for kidney injury
	Examples of these drugs: ACE inhibitors, NSAIDs, aminoglycosides, analgesic combinations, pentostatin, anti-angiogenesis medications, phenacetin, antivirals (such as acyclovir), ARBs, calcineurin inhibitors, hydroxyethyl starch, topiramate, ifosfamide, vancomycin, mTOR inhibitors, immunotherapies, zonisamide, ambisome, pamidronate disodium, amphotericin B, indomethacin, iodixanol (Visipaque), piperacillin/tazobactam, mesalamine, methotrexate, vancomycin, foscarnet, mitomycin, zoledronic acid, nafcillin, etc.
Naturally occurring nephrotoxins	Animal and insect venoms
	Kidney injury via various mechanisms: hemodynamic changes, microvascular thrombosis, AIN and direct cellular toxicity
	Botanicals cause kidney injury by intentional consumption as a food (usually in excess) or as part of traditional healing methods
	Examples: aristolochic acid, datura species, ephedra species, mefenamic acid, melamine, phenylbutazone, etc.
Environmental nephrotoxins	Daily threat, associated with many deaths worldwide
	Exposure through contaminated ground water, traditional medicines and soil remains a global health problem
	Exposure to heavy metals can cause tubular dysfunction acutely and progressive CKD with further exposure
	Drugs of abuse have nephrotoxic potential, including synthetic cannabinoids, MDMA, heroin and cocaine
	Other nephrotoxins: bismuth, cadmium, copper, hydrocarbons, lead, mercury, ethylene glycol poisoning from antifreeze ingestion, etc.

Abbreviations: ACE inhibitors—angiotensin-converting enzyme inhibitors; NSAIDs—non-steroidal anti-inflammatory drugs; ARBs—angiotensin receptor blockers; CKD—chronic kidney disease; AIN—acute interstitial nephritis; MDMA—3,4-methylenedioxymethamphetamine (ecstasy).

**Table 5:** Mechanisms of nephrotoxicity.

Mechanism of Nephrotoxicity	Special Features
Pseudo-nephrotoxicity	A fraction of serum creatinine is eliminated through active tubular secretion via several transporters, including OCT2
	Example: the drug trimethoprim, an OCT2 inhibitor, inhibits tubular secretion of creatinine, increasing its serum concentrations without direct injury to the kidney
Indirect nephrotoxicity	Medications (not directly nephrotoxic) contribute to systemic disease, causing kidney damage, such as hydroxy-methylglutaryl-coenzyme A (HMG-CoA) reductase inhibitors (statins)
	Example: rhabdomyolysis is a systemic syndrome that results in AKI due to vasoconstriction, direct ischemic injury, the proliferation of free radicals and tubular obstruction associated with myoglobinuria
Vascular complications	The kidney receives about 25% of the cardiac output and is sensitive to factors that influence systemic hemodynamics
	Excessive exogenous fluid administration may increase venous pressure in the kidney, leading to interstitial edema, which can reduce RBF and the GFR and lead to additional salt and water retention; fluid overload in critically ill children worsens outcomes
	Iatrogenic intravascular volume depletion (with drugs such as norepinephrine) due to excessive diuresis may decrease effective circulating arterial blood volume and compromise kidney perfusion, especially in patients with reduced baseline kidney perfusion
Acute interstitial nephritis	Cell-mediated immune response: drugs may act as haptens to modify the endogenous response to native renal proteins or induce an autoimmune reaction to the tubular basement membrane through molecular mimicry
	Drugs may also trigger systemic immune activation that leads to the deposition or sequestration of immune complexes in the kidney interstitium
	Interstitial infiltration by lymphocytes, macrophages, eosinophils and mast cells leads to inflammation and, eventually, fibrosis
	Kidney biopsy reveals interstitial inflammation, edema and tubulitis
	Drugs associated with AIN include beta-lactam antibiotics, rifampin, sulfonamides, NSAIDs, proton pump inhibitors and immune checkpoint inhibitors
Glomerular disease	Several drugs are associated with immune-mediated glomerular disease through the production of autoantibodies, such as ANCA-associated vasculitis with pauci-immune glomerulonephritis or drug-induced lupus
	Examples of these drugs: hydralazine, propylthiouracil, methimazole, cocaine, allopurinol and procainamide
	May also be due to direct cellular toxicity at the level of the podocyte, the endothelial cell or the mesangial cell that is caused by interferon, anabolic steroids, bisphosphonates and sirolimus
Direct tubular toxicity	Drugs or their metabolites may directly damage tubular cells via mitochondrial injury, oxidative stress or DNA damage, which can lead to apoptosis and necrosis
	Examples of these drugs: cisplatin, aminoglycosides, cidofovir, foscarnet, tenofovir and radiocontrast agents
Osmotic nephrosis	Drugs such as mannitol, hydroxyethyl starch, immunoglobulins and dextrans may lead to osmotic nephrosis through pinocytosis and cellular accumulation, leading to cell swelling, vacuolation and obstruction of the tubular lumen
Crystalluria/nephrolithiasis	Some medications or their metabolites are insoluble in the urine and can result in crystalline nephropathy and renal calculi formation
	Exacerbated by volume depletion, reduced urinary flow rates, high drug doses, rapid drug infusion rates and altered urine pH
	Examples of these drugs: acyclovir, methotrexate, sulfamethoxazole, indinavir, atazanvir, triamterene, ciprofloxacin and ethylene glycol
	Systemic conditions, such as tumor lysis syndrome, may also lead to crystal deposition in the kidney
Altered electrolyte handling	Drugs may alter kidney’s handling of phosphate, glucose, magnesium, potassium, sodium or water
	Specific patterns: SIADH, diabetes insipidus or acquired Fanconi syndrome
	Drugs causing this type of kidney dysfunction include lithium, ifosfamide, selective-serotonin reuptake inhibitors, anti-epileptics and vincristine

Abbreviations: OCT2—organic cation transporter; GFR—glomerular filtration rate; RBF—renal blood flow; NSAIDs—non-steroidal anti-inflammatory drugs; SIADH—syndrome of inappropriate antidiuretic hormone.

**Table 6:** Principles of supportive measures.

Parameter	Special Conditions	Description of Therapeutic Measures
*Fluid volume*	Hypovolemia	Immediate i.v. fluid administration in order to restore renal function and prevent progression to intrinsic AKI
	Euvolemia	Fluid losses (insensible fluid and urine GI losses) must be balanced with the given fluids
	Hypervolemia	Fluid removal/restriction needed
		Oliguric AKI: consider furosemide to convert AKI to nonoliguric form
		Early consideration for RRT in critically ill child
*Electrolytes*	Oliguria/anuria	Restriction of potassium and phosphorus
		Hyperkalemia is the most common electrolyte complication and is potentially life-threatening due to cardiac arrhythmia
		Therapy according to severity of hyperkalemia
	Sodium	Intake restriction to 2–3 mEq/kg/day to prevent fluid retention and hypertension
	Polyuria	Replacement of electrolyte losses
*Acid–base balance*	Metabolic acidosis	Common abnormality of AKI
		NaHCO_3_ indicated in life-threatening situations despite possible adverse effects
*Hypertension*		Common complication of AKI
		Therapy according to the severity and cause of hypertension
*Nutrition*		AKI is associated with catabolism
		Nutritional support needed to enhance the recovery
		Normal nutritional maintenance requirements and supplemental calories to address the catabolic needs
		Caloric intake: at least 120 Kcal/kg/day in infants and at least 150% of maintenance needs in older children
*Drugs*		Avoidance of nephrotoxic agents
		Dosing adjustment of renally excreted drugs according to renal function

Abbreviations: AKI—acute kidney injury; GI—gastrointestinal; NaHCO_3_—sodium bicarbonate.

## Data Availability

Not applicable.
